# Global Health Governance and the Commercial Sector: A Documentary Analysis of Tobacco Company Strategies to Influence the WHO Framework Convention on Tobacco Control

**DOI:** 10.1371/journal.pmed.1001249

**Published:** 2012-06-26

**Authors:** Heide Weishaar, Jeff Collin, Katherine Smith, Thilo Grüning, Sema Mandal, Anna Gilmore

**Affiliations:** 1 Centre for Population Health Sciences, University of Edinburgh, Edinburgh, United Kingdom; 2 Global Public Health Unit, University of Edinburgh, Edinburgh, United Kingdom; 3 Department for Health, University of Bath, Bath, United Kingdom; San Diego State University, United States of America

## Abstract

**Background:**

In successfully negotiating the Framework Convention on Tobacco Control (FCTC), the World Health Organization (WHO) has led a significant innovation in global health governance, helping to transform international tobacco control. This article provides the first comprehensive review of the diverse campaign initiated by transnational tobacco corporations (TTCs) to try to undermine the proposed convention.

**Methods and Findings:**

The article is primarily based on an analysis of internal tobacco industry documents made public through litigation, triangulated with data from official documentation relating to the FCTC process and websites of relevant organisations. It is also informed by a comprehensive review of previous studies concerning tobacco industry efforts to influence the FCTC. The findings demonstrate that the industry's strategic response to the proposed WHO convention was two-fold. First, arguments and frames were developed to challenge the FCTC, including: claiming there would be damaging economic consequences; depicting tobacco control as an agenda promoted by high-income countries; alleging the treaty conflicted with trade agreements, “good governance,” and national sovereignty; questioning WHO's mandate; claiming the FCTC would set a precedent for issues beyond tobacco; and presenting corporate social responsibility (CSR) as an alternative. Second, multiple tactics were employed to promote and increase the impact of these arguments, including: directly targeting FCTC delegations and relevant political actors, enlisting diverse allies (e.g., mass media outlets and scientists), and using stakeholder consultation to delay decisions and secure industry participation.

**Conclusions:**

TTCs' efforts to undermine the FCTC were comprehensive, demonstrating the global application of tactics that TTCs have previously been found to have employed nationally and further included arguments against the FCTC as a key initiative in global health governance. Awareness of these strategies can help guard against industry efforts to disrupt the implementation of the FCTC and support the development of future, comparable initiatives in global health.

*Please see later in the article for the Editors' Summary*

## Abstract 2

Heide Weishaar and colleagues did an analysis of internal tobacco industry documents together with other data and describe the industry's strategic response to the proposed World Health Organization Framework Convention on Tobacco Control.

## Editors' Summary

**Background:**

Every year, about 5 million people die worldwide from tobacco-related causes and, if current trends continue, annual deaths from tobacco-related causes will increase to 10 million by 2030. In response to this global tobacco epidemic, the World Health Organization (WHO) has developed an international instrument for tobacco control called the Framework Convention on Tobacco Control (FCTC). Negotiations on the FCTC began in 1999, and the international treaty—the first to be negotiated under the auspices of WHO—entered into force on 27 February 2005. To date, 174 countries have become parties to the FCTC. As such, they agree to implement comprehensive bans on tobacco advertising, promotion, and sponsorship; to ban misleading and deceptive terms on cigarette packaging; to implement health warnings on tobacco packaging; to protect people from tobacco smoke exposure in public spaces and indoor workplaces; to implement taxation policies aimed at reducing tobacco consumption; and to combat illicit trade in tobacco products.

**Why Was This Study Done?:**

Transnational tobacco corporations (TTCs) are sometimes described as “vectors” of the global tobacco epidemic because of their drive to maximize shareholder value and tobacco consumption. Just like conventional disease vectors (agents that carry or transmit infectious organisms), TTCs employ a variety of tactics to ensure the spread of tobacco consumption. For example, various studies have shown that TTCs have developed strategies that attempt to limit the impact of tobacco control measures such as the FCTC. However, to date, studies investigating the influence of TTCs on the FCTC have concentrated on specific countries or documented specific tactics. Here, the researchers undertake a comprehensive review of the diverse tactics employed by TTCs to undermine the development of the FCTC. Such a review is important because its results should facilitate the effective implementation of FCTC measures and could support the development of future tobacco control initiatives and of global initiatives designed to control alcohol-related and food-related disease and death.

**What Did the Researchers Do and Find?:**

The researchers analyzed documents retrieved from the Legacy Tobacco Documents Library (a collection of internal tobacco industry documents released as a result of US litigation cases) dealing with the strategies employed by TTCs to influence the FCTC alongside data from the websites of industry, consultancy, and other organizations cited in the documents; the official records of the FCTC process; and previous studies of tobacco industry efforts to influence the FCTC. Their analysis reveals that the strategic response of the major TTCs to the proposed FCTC was two-fold. First, the TTCs developed a series of arguments and “frames” (beliefs and ideas that provide a framework for thinking about an issue) to challenge the FCTC. Core frames included claiming that the FCTC would have damaging economic consequences, questioning WHO's mandate to develop a legally binding international treaty by claiming that tobacco was not a cross-border problem, and presenting corporate social responsibility (the commitment by business to affect the environment, consumers, employees, and society positively in addition to making money for shareholders) as an alternative to the FCTC. Second, the TTCs employed multiple strategies to promote and increase the impact of these arguments and frames, such as targeting FCTC delegations and enlisting the help of diverse allies including media outlets and scientists.

**What Do These Findings Mean?:**

These findings illustrate the variety and complexity of the tobacco industry's efforts to undermine the FCTC and show the extent to which TTCs combined and coordinated tactics on a global stage that they had previously used on a national stage. Indeed, “the comprehensiveness and scale of the tobacco industry's response to the FCTC suggests that it is reasonable to speak of a ‘globalisation of tobacco industry strategy’ in combating the development of effective tobacco control policies,” write the researchers. Awareness of the strategies employed by TTCs to influence the FCTC should help guard against industry efforts to disrupt the implementation of the FCTC and should support the development of future global tobacco control initiatives. More generally, these findings should support the development of global health initiatives designed to tackle cardiovascular disease, cancer, chronic respiratory diseases and diabetes – non-communicable diseases that together account for 60% of global deaths and are partly driven by the commercial activities of food, alcohol, and tobacco corporations.

**Additional Information:**

Please access these Web sites via the online version of this summary at http://dx.doi.org/10.1371/journal.pmed.1001249.

- The World Health Organization provides information about the dangers of tobacco (in several languages) and about the Framework Convention on Tobacco Control
- For information about the tobacco industry's influence on policy, see the 2009 World Health Organization report Tobacco interference with tobacco control
- The Framework Convention Alliance provides more information about the FCTC
- The Legacy Tobacco Documents Library is a public, searchable database of tobacco company internal documents detailing their advertising, manufacturing, marketing, sales, and scientific activities
- The UK Centre for Tobacco Control Studies is a network of UK universities that undertakes original research, policy development, advocacy, and teaching and training in the field of tobacco control
- SmokeFree, a website provided by the UK National Health Service, offers advice on quitting smoking and includes personal stories from people who have stopped smoking
- Smokefree.gov, from the US National Cancer Institute, offers online tools and resources to help people quit smoking and not start again

## Introduction

The WHO Framework Convention on Tobacco Control (FCTC), the first international public health treaty initiated by the World Health Organization (WHO), arguably represents the most significant tobacco control initiative to date and has been central to WHO's efforts to reestablish its strategic significance. The treaty constitutes a landmark in global health governance [1], characterised by complex “worldwide transboundary interactions” and a recognition that global health is shaped by international organisations, corporations, philanthropists, and civil society, as well as nation states [2],[3] and marks an ambitious and innovative response to the global tobacco epidemic.

In their drive to maximise shareholder value and global tobacco consumption, transnational tobacco corporations (TTCs) have been described as a “vector” of this epidemic [4]. Research to date has revealed a variety of tactics designed to help TTCs achieve their goals, including efforts to limit the FCTC's impact. Articles focusing on TTCs' influence on the FCTC have, however, principally focused on country- or issue-specific case studies and on documenting specific tactics. In contrast, this paper provides the first comprehensive review of TTCs' use of global-level tactics to undermine the development of the FCTC. The findings will enhance understanding of obstacles to the effective implementation of FCTC measures and to the negotiation of FCTC protocols, and make a contribution to both the development of future tobacco control initiatives and any comparable global initiatives in related health issues [5],[6],[7]. The paper will inform ongoing debates about the role of corporations in health policy development and global governance, particularly in terms of international agencies' responses to the global burden of non-communicable diseases (NCDs) [8].

## Methods

This paper is based on analysis of previously confidential tobacco industry documents made publicly available through litigation in the United States and is informed by a comprehensive review of the existing literature concerning TTCs' strategies to influence the FCTC. Online searches of the Legacy Tobacco Documents Library (http://legacy.library.ucsf.edu) were conducted between May 2008 and February 2009 to identify relevant documents. Preliminary searches focused on broad terms (e.g. “Framework Convention on Tobacco Control”, “FCTC” and “WHO Convention”), which informed more specific searches (for particular names, issues, and events). Searches were undertaken in English (the language in which the majority of the documents are written) and German (reflecting Germany's reputation as a key tobacco industry ally [9]). In total, over 4,500 documents were reviewed, of which 1,366 documents were deemed relevant. These documents were analysed in detail and indexed using EndNote. This paper draws on a selection of these (84 from British American Tobacco [BAT], 28 from Philip Morris [PM], five from RJ Reynolds, and two from Brown and Williamson).

A qualitative, hermeneutic methodology was employed to analyse the documents [10],[11], and the thematic coding framework was developed inductively (employing two major categories and multiple subcategories). Alongside the thematic analysis, the documents were organised chronologically to construct a historical narrative. This analysis was contextualised with additional data from the websites of industry, consultancy, and other organizations cited in the documents and official FCTC documentation. Official documentation from the negotiations was accessed via the WHO FCTC website (http://www.who.int/fctc/en/) and was supplemented by reports from 45 Framework Convention Alliance (FCA) Bulletins, published at the sessions of the International Negotiation Body (INB) (http://www.fctc.org/).

Regarding the comprehensive review of the existing published literature concerning tobacco industry efforts to influence FCTC negotiations, systematic searches were conducted using the search terms “Framework convention on tobacco control” OR (FCTC AND tobacco) between November 2011 and January 2012 in the following databases: Global Health (CABI, 172 hits), PubMed (140 hits), Web of Knowledge (119 hits), the Centre for Tobacco Control Research and Education's e-scholarship website (http://escholarship.org/uc/ctcre) (96 hits), Applied Social Sciences Index and Abstracts (ASSIA, 50 hits), and the International Bibliography of the Social Sciences (IBSS, 13 hits). A total of 590 articles were retrieved. All abstracts were checked and duplicates were excluded. Of these, 154 articles were deemed potentially relevant, so full versions were accessed to allow a detailed review. Relevant articles were selected according to the following inclusion criteria: written in English, informed by empirical research (i.e., not just opinion pieces), and focused on tobacco industry tactics and arguments to influence the FCTC. These criteria were met by 34 articles. The results from this review provided background and comparative information for our documentary analysis and are drawn on in both the results and the discussion.

The methodological approach of the study was approved by the Research Ethics Committee of the University of Bath's School for Health.

## Results

Our literature review identified 12 country-specific case studies showing, for example, how TTCs tried to use their influence on German politicians to weaken the FCTC [12], cooperated with the Japanese delegation [13], and managed to obstruct the implementation of the FCTC in Argentina [14]. A further 22 studies focused on the pursuit of specific tactics, e.g., the development of voluntary regulation [15] or the use of consultancies [16]. Only ten articles were based on primary research. Many of the studies identified through this review recognised that the tactics described existed within a broader comprehensive global industry strategy to undermine the FCTC negotiations [12],[15],[17],[18], but none sought to address the complexity of such a strategy. Hence, this paper represents the first comprehensive analysis of the extent of TTCs' tactics to influence the FCTC.

Our documentary analysis revealed that all major TTCs were considerably alarmed about the FCTC from its initiation [19],[20],[21],[22] and recognised the need to respond comprehensively [23],[24],[25],[26],[27]. This reflected concern about the scope of the convention, the “breathtaking” [22] progression of developments, and their potential catalytic effects on national and regional tobacco control regulation [22]. Having rapidly identified the proposed convention as an “unprecedented challenge to the tobacco industry's freedom to continue doing business” [19], it is unsurprising that TTCs responded aggressively to this use of WHO's constitutional authority. The account below focuses particularly on BAT's strategy to counter the FCTC, reflecting the balance of available documents, BAT's self-proclaimed status as the most international tobacco group [28], and the company's strengths in developing countries, where industry interests were perceived as most threatened by the initiative [29].

The Results section is divided into two subsections which outline the frames and tactics TTCs employed in an FCTC context, respectively. Both sections focus primarily on the documentary data, but the findings from the literature review are incorporated into Tables 1 and 2 and are drawn on in the textual discussion where relevant. As it would be impossible to describe all of the activities relating to each strategy within one paper, the following sections use examples to illustrate the frames and tactics identified.

**Table 1:** Tobacco industry frames to influence the FCTC.

Frame	Goal	Evidence of Application
***TTC frames identified through the analysis of tobacco industry documents and a comprehensive literature review***		
1. *Economic impacts*: Alleging damaging economic consequences of the FCTC	To depict tobacco control as detrimental to the economy and threaten policy makers and politicians; particularly effective during economic recession	[12],[17],[36],[229]
2. *Developing countries*: Depicting tobacco as a high-income country issue	To divert attention away from tobacco control; to cause and increase dissent and hostility	[17],[36],[230],[231],[232]
3. *Trade*: Claiming conflicts with trade agreements	To stall tobacco control initiatives, including regulation of tobacco ingredients, health warning labels, plain packaging, etc.	[17],[233]
4. *WHO's mandate*: Questioning WHO's mandate to develop a tobacco control treaty	To question the legal basis for tobacco control initiatives and particularly prevent legislation spanning across national and regional borders	[12],[17]
5. *Good governance*: Alleging conflicts with principles of good governance and national sovereignty	To increase opposition against tobacco control and the policy makers responsible for it; to stall the process of tobacco control policy making	[230]
6. *Sovereignty*: Alleging conflicts with national sovereignty	To make the case that international tobacco control undermines national sovereignty, including by questioning the legitimacy of international tobacco control and in so doing, to raise opposition of nation states	[12],[13],[230],[234]
7. *Corporate social responsibility*: Presenting CSR as an alternative to tobacco control policy	To create an illusion of being a “changed,” more socially responsible company; to regain political and public credibility	[15],[96]
***TTC frames identified through the analysis of tobacco industry documents only***		
8. *Precedent*: Depicting the FCTC as setting a precedent for other areas	To enlist allies in debates by claiming relevance to other health issues and potential implications for other industries	
***TTC frames identified through a comprehensive literature review only***		
9. *Legal product:* Stressing that tobacco is a legal product	To stress that tobacco is a legal product which should be treated like any other issue	[235]
10. *Flexibility:* Stressing that tobacco control should be “flexible” and “appropriate”	To depict stringent tobacco regulation as rigid and unreasonable	[12],[13]
11. *Extremism:* Depicting tobacco control advocates are extremist, radical, and not credible	To portray tobacco control advocates and their positions as unacceptable and, by contrast, tobacco industry positions as moderate and reasonable	[16],[230]
12. *Personal freedoms:* Claiming that tobacco control infringes on freedom of expression and other personal freedoms, and states that pass tobacco control are “nanny states”	To maintain the tobacco industry's ability to market their products; and to maintain the social acceptability of smoking	[12]
13. *Harmlessness:* Claiming that tobacco is not harmful to health or its effect is minimal	To cast doubt on the scientific evidence that smoking is health damaging and play down the seriousness of the health problem	[230]
14. *Education:* Focusing on youth smoking prevention and retailer education	To appear to help prevent underage smoking and to depict smoking as an adult choice, although research suggests industry-sponsored programmes are usually ineffective (often linked to CSR programmes)	[15],[16],[230],[236]

**Table 2:** Tobacco industry tactics to influence the FCTC.

Tactic	Related Goals	Evidence of Application
***Industry FCTC tactics identified through the analysis of corporate documents and a comprehensive literature review***		
1. Targeting national FCTC delegations and political actors (via lobbying and infiltration of organisations and committees with influence)	• To promote particular ideas and information, attempt to make deals, and generally influence political processes• To persuade policymakers that tobacco control proposals conflict with other, existing legislation (such as trade agreements)• To infiltrate decision-making bodies and influence political decisions• To mobilise decision makers with opposing views in order to increase opposition against tobacco control legislation and influence political debates and decisions• To preempt FCTC legislation by passing TTC favoured regulation with the aim of forestalling or delaying stronger regulation	[12],[13],[16],[17],[36],[229],[230],[237],[238],[239],[240],[241],[242],[243],[244],[245]
2. Use of scientists	To create doubt and undermine evidence about the negative impacts of tobacco use and the efficacy of tobacco control measures	[17],[233]
3. Enlisting and mobilising allies (including other industry sectors, umbrella business organisations, trade unions, international agenciesand other political actors)	• To enhance the credibility of tobacco industry campaigns• To create an impression of spontaneous, grassroots public support for particular (TTC favoured) positions• To provide advice to TTCs or to lend credibility to positions favoured by TTCs	[12],[16],[17],[18],[36],[229],[230],[233],[236]
4. Using stakeholder consultation to secure industry participation and delay decisions	• To ensure tobacco TTC participation and representation in policy discussions• To facilitate agenda setting and tobacco industry influence throughout political discussions• To gain time to frame debates, implement other tactics, and continue to make profits	[12],[16],[230],[235]
5. Using the media	• To influence public opinion• To promote positions favourable to the industry	[17]
***TTC tactics identified through a comprehensive literature review but not evident in our analysis of corporate documents***		
6. Countering nongovernmental organisations	To fight and weaken opposition against TTCs, discredit those who challenged the TTCs' positions, and divide the tobacco control community	[16],[230],[241],[246],[247]
7. Intimidation	To use legal and economic power or arguments as a means of harassing and frightening supporters of tobacco control and threaten policymakers that they will lose elections	[12],[14]
8. Obstructing ratification and effective implementation of tobacco control	To dilute and neutralise the effect of tobacco control legislation	[14],[229],[238],[244],[248],[249],[250],[251],[252]
9. Roadshow-type activities	To shift the public opinion and debate	[17]
10. Achieving joint manufacturing and licensing agreements and policy agreements with governments	To form joint ventures with state monopolies to gain market share and subsequently pressure governments to privatize monopolies	[15],[16],[230]

### 

#### (A) Attempts to frame debates and develop argumentation against the FCTC

The concept of “framing” describes a strategy based on generating beliefs and ideas that provide a framework for thinking about an issue [30],[31]. Policy frames have been described as a “weapon of advocacy” [32],[117] which, when successfully applied, can be crucial in shaping policy [33]. Schattenschneider [34] argues that the definition of political issues and potential solutions determines who is involved in the policy process and shapes both the organisation of interests and the formation of coalitions and alliances. Our searches showed that numerous potential ways of framing debates around tobacco control and international regulation were explored by TTCs. We, however, identified eight core frames that were used in relation to the FCTC negotiations, which sometimes overlap and are mutually reinforcing, but nevertheless exhibit distinctive features. These eight frames are outlined in the following list. A further six frames were identified in our comprehensive literature review of publications relating to the FCTC. All 14 frames are presented in Table 1 and returned to in the Discussion.

(1) *Economic impacts: Alleging damaging economic consequences of the FCTC.*

In 1999, a World Bank report demonstrated that economic fears of tobacco control legislation having negative impacts were largely unfounded, and that tobacco control policies would benefit most national economies [35]. The World Bank backed the FCTC initiative and encouraged governments to employ comprehensive strategies to curb the epidemic [35]. Nevertheless, the tobacco industry adopted arguments predicting the FCTC would cause economic harm, including via lost wages for tobacco farmers, reduced employment opportunities (particularly in rural areas), and lost tobacco crop revenue. Such arguments had already been used to counter national tobacco control initiatives [14],[36] but were now reiterated to raise concerns about the FCTC with specific member states, including Italy, Greece and Turkey, Brazil, Argentina, Zimbabwe, India, and Russia [37],[38],[39]. It was alleged that the FCTC would be particularly detrimental to tobacco growing in developing countries. A key role in promoting such arguments was played by the International Tobacco Growers Association (ITGA) [19], which was formed by TTCs [40], served on several occasions as a frontgroup for TTCs [41],[42],[43] and still receives funding from PM, BAT, and Imperial Tobacco International [44]. The ITGA proclaimed a need to “take into account the very real impact of [the FCTC] upon farms, their families, communities and national economies” [45] and, in cooperation with Europe's International Union of Tobacco Growers (UNITAB), depicted the FCTC as an “initiative which can have desastrous [*sic*] consequences for millions of people in the world, who depend on tobacco growing for their living” [46].

In an attempt to highlight the FCTC's alleged economic impacts, BAT tried to alert national governments “to the costs of a WHO tobacco police state” [47], suggesting that international organisations can “take on a life of their own, demanding (and sometimes wasting) large contributions from taxpayers” [48] and imposing bureaucratic [48] and other “new burdens on governments” [49].

(2) *Developing countries: Depicting tobacco as a high-income country issue.*

Notwithstanding the rapidly escalating health and economic burdens of tobacco use in developing countries [50], this industry frame depicted tobacco control as an issue primarily of concern to high-income countries. A key architect of this frame was Roger Bate, an economist of long-standing tobacco industry affiliation [51],[52],[53] and founder of the European Science and Environmental Forum (ESEF), a think-tank that the tobacco industry sought to establish as a “scare watchdog” [54] and use to raise debates about scientific evidence [51],[54],[55]. Bate suggested assembling academics working on malaria to “create tensions between LDCs [less-developed countries] and OECD countries and between public health [i.e. communicable diseases] and environment [including non-communicable diseases]” [56]. He argued that “we can divide our opponents and win” by showing them “where their alleged allies are harming their cause” [56]. Bate's suggestions were positively received at PM [57]. A 1999 book entitled “Environmental Health: Third World Problems - First World Preoccupations” by Bate and Lorraine Mooney, an ESEF colleague, argued that communicable diseases were the primary health problem confronting developing countries and therefore the most appropriate focus for WHO [58]. Bate disseminated these claims via the international media, including in a letter to the Financial Times in October 2000 (coinciding with the opening of formal FCTC negotiations), in which he claimed that the FCTC would undermine “the sovereignty of nations” and called on “national governments to reject the Convention in its current form” [59].

Such arguments allowed TTCs to portray the FCTC as a neocolonial initiative that would benefit richer nations at the expense of poorer ones. For example, BAT's then-chairman, Martin Broughton, used a speech at the 1999 World Economic Forum to redirect public attention to what he termed the “real issues in the developing world like malnutrition, sanitation and infant mortality” [60]. Similarly, the German Cigarette Manufacturers Association, the Verband der Cigarettenindustrie (VdC), issued a press statement alleging that WHO's work towards the convention demonstrated “disrespect for the real needs of the poor of this world” [61].

(3) *Trade*: *Claiming conflicts with trade agreements.*

TTCs also sought to depict the proposed FCTC as inconsistent with obligations under existing international agreements, notably those of the World Trade Organisation (WTO). Such arguments were central to negotiations, and tensions between public health and trade policies were widely discussed by academics, advocates, and officials [62],[63],[64]. Academics argued that FCTC measures could be adopted without impacting on free trade rules [65], and a joint report by WTO and WHO in 2002 highlighted that none of the FCTC proposals were “inherently WTO-inconsistent” [66]. Nevertheless, TTCs and the VdC commissioned multiple legal analyses of the issue [67],[68],[69],[70] to support their claims that conflicts between FCTC proposals and existing international trade agreements existed (the contents of these analyses have largely been withheld from public disclosure on the basis of attorney-client privilege) [71]. Internal PM correspondence shows that PM commissioned the law firm Beveridge and Diamond to “prepare a memorandum focusing on the potential trade policy implications of the Convention [assessing] recent Conventions in other arenas and identify[ing] instances where WTO or other trade principles created both jurisdictional and substantive conflicts” [69]. A letter to Andreas Vecchiet (BAT International Political Affairs Manager) from Crowell & Moring International, an international policy and regulatory affairs consulting firm, suggests BAT could adopt a similar strategic approach to manage trade issues [67]. The firm suggested an analysis of “the extent to which the proposed Framework will raise inconsistencies with countries' WTO obligations” and offered to help BAT to develop “strategies to engage trade and agricultural officials” in the FCTC negotiations [67]. BAT subsequently argued that FCTC proposals would violate international trade laws [72],[73], a position supported by German trade and industry associations [74]. The company contracted the public affairs firm Prisma to draft “short and fairly basic” briefing papers to be “used by ministers (and officials)” which would claim that “WHO provisions contravene articles X, Y and Z of the WTO” [70].

(4) *WHO's mandate*: *Questioning WHO's mandate to develop a tobacco control treaty.*

TTCs questioned WHO's authority and competence to develop a legally binding international treaty, claiming that tobacco was “not a ‘cross-border’ problem” [24] and so should not be dealt with at a global level [23]. Although two legal analyses commissioned by BAT acknowledged that WHO was competent to negotiate a tobacco control convention [76],[77], the company continued to raise questions about WHO's mandate [23]. In a September 1998 correspondence with PM Vice President of corporate affairs, David Greenberg, Bate successfully proposed a series of papers to frame debates around WHO's inadequate priority setting [56]. Similarly, an analysis by the legal consultancy firm Rowe and Maw, possibly intended for publication at a conference on international health policy [104], portrayed several FCTC proposals as extending “well beyond the core areas of authority contemplated by the WHO constitution, raising legal issues of whether measures taken under the auspices of the WHO […] are within the powers of the WHO” [75]. BAT further obtained an analysis of policy implications that could be raised by countries wanting to dispute WHO's authority [78].

(5) *Good governance: Alleging conflicts with principles of good governance.*

A variation on questioning WHO's mandate was to attack the FCTC process as infringing principles of “good governance” and “sensible regulation” [23]. BAT used these terms to signify the company's preferred mix of nonbinding national agreements and voluntary measures [79] and to promote policymaking frameworks which drew attention to business interests [80],[81]. The company used this frame to claim that tobacco industry representatives were being unfairly excluded from FCTC negotiations [19],[39],[82],[83], and alleged that this “unprecedented failure to consult” [84] was contrary to “good public policy formation” [23], neglected some expert opinions and facilitated biased discussions [78].

(6) *Sovereignty*: *Alleging conflicts with national sovereignty.*

BAT also depicted WHO, an international organisation, as essentially undemocratic [25] and representing a transfer of power to “unaccountable and remote elites” [24], and described the FCTC as a “one-size-fits-all” approach [72], which was “bound to fail” [75]. This was contrasted against national legislation, which was depicted as carefully developed and locally sensitive [75]. BAT sought to persuade WHO member states that the FCTC would infringe their sovereign rights to legislate in areas of tobacco control [23]. Accordingly, BAT's legal department, in cooperation with the international law firm August and Debouzy [85], analysed national constitutions to identify conflicts with the FCTC [86] and subsequently sought to convince governments that ratifying such a treaty could restrict options for national legislation [38],[84],[87].

(7) *Corporate social responsibility (CSR): Presenting CSR as an alternative to a convention.*

In line with their broader emphasis on CSR [88],[89],[90], TTCs sought to present themselves as responsible corporate citizens throughout the FCTC process. Such attempts were intended to achieve three main aims: (i) to raise the companies' profile [91],[92]; (ii) to facilitate access to relevant policymakers and discussions by improving their perceived credibility [15],[93],[94]; and (iii) to avoid strong and binding legislation by presenting self-regulation as an effective alternative [15]. These aims were promoted via diverse CSR initiatives, including a conference on eliminating child labour in tobacco-growing countries organized by BAT, ITGA, and the International Union of Food, Agricultural, Hotel, Restaurant, Catering, Tobacco and Allied Workers' Associations (IUF). The conference was designed to enhance the “ITGA's relationship in the UN arena” [94] and increase BAT's “recognition as a responsible company” [95],[96]. The ITGA also publicised grower-funded programmes to combat AIDS in Africa, hoping to advance the *developing countries* frame, refocus attention on other health issues, and discredit WHO [91]. TTCs also collaborated in developing a global voluntary code on youth smoking prevention which was intended to be an alternative to the FCTC [15].

The use of CSR initiatives and voluntary measures aimed to position TTCs as credible and legitimate stakeholders who merited inclusion in discussions [90]. Industry documents indicate that TTCs used commitments to voluntary measures to demonstrate a willingness to “participate constructively” [97] in international health policy [25],[72],[98],[99]. While such approaches seem to have had some success in terms of positioning TTCs as legitimate stakeholders in national policy debates [90], available documents do not indicate that they had a substantive impact on international FCTC negotiations.

(8) *Precedent: Depicting the FCTC as setting a precedent for other areas.*

BAT also sought to expand the “threat” posed by the FCTC beyond tobacco, depicting the convention as part of broader, “worrying anti-business trends that many companies have identified within the UN and multi-lateral system” [100]. BAT claimed the FCTC could set a dangerous precedent for other industries [101] and that, in developing the FCTC, the WHO was “acting as the world's ‘super nanny’” [102]. TTCs further sought to emphasize the potential ramifications of such an instrument based in international law for other industries [101],[103]. In February 2000, Rowe and Maw advised BAT “to expand the scope of the debate to cover other industries […] and to raise the debate to a higher level of generality” [103]. They suggested holding a conference on international health policy to debate the WHO's mandate (*WHO's mandate* frame) and its impact on national sovereignty in the regulation of alcohol, tobacco, and pharmaceuticals (*sovereignty* frame) [104]. A memo in March 2001, by BAT's Nicola Shears (formerly of the UK department of trade and industry [105]), suggests this tactic may have met with some success, as it reported that “other sectors are watching the WHO's activity with increasing concern over the WHO's apparent enthusiasm to become the ‘global health police’” [101].

#### (B) Strategic efforts

TTCs also employed tactics to actively influence the development of the FCTC, including efforts to effectively disseminate the frames and arguments outlined above. We identified five such tactics in our documentary analysis, each of which is outlined below and a further five tactics in our comprehensive literature review of publications relating to the FCTC. All ten tactics are outlined in Table 2.

(1) *Targeting national FCTC delegations and political actors.* TTCs targeted the political actors involved in the international FCTC negotiations, making a particular effort to target actors perceived to be both powerful and amenable to tobacco industry positions. An analysis conducted for PM by Mongoven, Biscoe & Duchin (MBD), a consultancy firm with longstanding links to PM [16], adopted a classification by Porter et al. [106], categorising national governments' position in relation to the FCTC as “lead states,” “supporting states,” “swing states,” and “veto or agenda weakening states” [107]. TTCs subsequently directed their efforts to states identified as amenable to industry arguments [21],[108],[109], agreeing that “homing in on specific governments […] and regions” [110] was necessary to “[m]aintain and enhance activities of key governments” [111]. Within Europe, PM initially identified Germany, Denmark, Turkey, and France as potentially supportive [112] but, following intense industry lobbying, the German [12],[113], Spanish [114], Turkish [115],[116], and Russian [117] delegations seemed to emerge as the most useful allies. BAT documents repeatedly mention these four states as “key countries” [118],[119],[120] that could help raise concerns about the FCTC [108].

Building on various frames (e.g., *developing countries* and *trade*), TTCs hoped to create conflicts between delegations by exploiting varying national interests and mobilising supportive governments to “facilitate coalitions of like-minded countries” in opposition to the FCTC [111]. MBD noted that “proposals can be surfaced which assist many developing countries but which seriously harm others. Resolution of such issues is time consuming and often embittering” [107]. Documents indicate that efforts to create controversy between delegations [107],[121] may have had some impact on the conduct of FCTC negotiations [122],[123],[124],[125], with Germany and Russia playing prominent roles in these debates. Germany reportedly exerted influence on European and Latin American countries to weaken the FCTC [12], while Russia was seen as capable of influencing other former Soviet Union countries and of building an alliance with China [126]. During the discussions of the INB, both Russia and Turkey rejected suggested measures to give the FCTC precedence over trade agreements in the event of conflicts arising [123],[127].

TTCs also sought to stimulate inter-ministerial conflicts within governments [12] by encouraging the involvement of economic, justice, and trade ministries [38],[128],[129] as well as health ministries [120]. This tactic, previously used to influence national and regional tobacco control [14],[130],[131], aimed to promote less-stringent, voluntary agreements as viable alternatives to the convention. Within TTCs, this approach was viewed as successful in the UK [108], Germany [12], and Japan [13],[132] and as an effective strategy to deflect debates and decisions at supra- and international levels [12].

A related tactic was to push national governments to implement weak legislation, in an effort to preempt more stringent regulation arising from FCTC ratification. Preemption measures apparently met with some success in Mexico [133] and Argentina [14],[134]. In Argentina, two resolutions were passed following lobbying by the Argentinean tobacco industry [14] that ensured that no international treaty could be signed which would regulate local tobacco production and consumption [134]. While Argentina signed the FCTC on 25 September 2003, it has yet to ratify the treaty.

(2) *Use of scientists.* TTCs engaged scientists to advance their arguments and frames, an established industry tactic designed to shape scientific discourse and public opinion [17],[43],[131],[135],[136]. In December 1999, Beveridge and Diamond identified the assessment of the “possible role of industry experts and academic/policy fora to shape substantive debate” as one action that PM could take with regard to the FCTC [137]. PM intended to use scientists and other experts to support several of the frames outlined above: tensions with national sovereignty (*sovereignty* frame), the economic importance of tobacco (*economic impacts* frame) [26], and the primacy of other health priorities (*developing countries* frame) [56]. The ITGA targeted academics who could be persuaded to focus on health issues in developing countries (*developing countries* frame) [138] and proposed a conference in the UK to discuss the socioeconomic importance of tobacco (*economic impacts* frame) [139]. Roger Scruton, a British conservative philosopher, was commissioned by Japan Tobacco to denounce international bureaucracy, with a specific focus on the FCTC (*good governance* frame) [140],[141],[142], and TTCs hired academics to counter the World Bank's work and to disseminate research on the FCTC's allegedly detrimental economic consequences (*economic impacts* frame) [17].

(3) *Enlisting and mobilising allies.* Conscious of their own declining credibility [90], TTCs attempted to engage the support of diverse allies from other industry sectors, industry associations, international agencies, and several front groups to lend credibility to the frames and arguments outlined in section A above.

Enabling “credible third part[ies]” to support the campaign against the Tobacco Free Initiative was seen as essential [139] by PM, since such third parties could “be more aggressive in opposing [the] FCTC” [26]. One rationale for cooperation amongst TTCs was to maximise their ability to mobilise non-tobacco allies [111]. Conflicts did, however, emerge between PM and the other companies [62], as PM publicly presented itself as a responsible company broadly sympathetic to the FCTC [98],[143], whilst the other TTCs remained firmly opposed. Indeed, PM's above-mentioned tactic of enlisting allies who could militantly oppose the convention is particularly striking, as it highlights the discrepancy between the company's public statements (in which PM emphasised “common ground” with WHO, public health officials, and proponents of the FCTC [98],[143]) and its covert actions.

*Other industry sectors*: TTCs' cooperation with retailers [113],[144],[145],[146],[147], and with hospitality [147], advertising [111] and duty-free [148] industries ensured that the FCTC was attacked from diverse quarters and perspectives. BAT, for example, collaborated with international duty-free interest representatives (including travel associations and airports) [111] and advertising associations, using established allies like the International Advertising Association (IAA) [120]. Both sectors made submissions to the FCTC public hearings with, for instance, the German Duty Free Confederation reiterating tobacco industry positions [12],[149],[150]. The IAA [151] and the German Advertising Foundation [152] argued that a comprehensive advertising ban would violate freedom of speech, be unconstitutional, infringe human rights, and threaten competition [151],[152]. A submission by the Advertising Council of Russia, prepared by BAT Russia [153], also alleged detrimental economic impacts (*economic impacts* frame) [154].

*Umbrella business organisations*: BAT also anticipated benefits from working with organisations representing broader business interests, such as the Russian national trade association [153] and the Union of Industrial and Employers' Confederations of Europe (UNICE, now rebranded BusinessEurope) [70], with whom BAT had previously collaborated to achieve regulatory change in the EU [81]. BAT attached particular significance to encouraging the International Chambers of Commerce (ICC) to voice concerns (see Box 1).

**Box 1. BAT and the ICC**

In autumn 1999, Broughton accepted an invitation to join the ICC UK Governing Body [223]. In its invitation, the ICC noted that they had past experience of supporting tobacco companies in the fight against tobacco control [224]. BAT believed the ICC provided “a neutral platform,” which would enable BAT “to access key stakeholders in the UK and internationally” [225]. The ICC's status as “a truly global body representative of industry views” facilitated engagement in debates and lobbying activities at an international level [223], including in the WTO [225]. In the context of the FCTC, the ICC was perceived to be an “an important stakeholder which we can really leverage if we get it right” [226]. BAT hoped that the ICC would act as the “business and industry's ears” in FCTC negotiations [227], monitoring and reporting on its behalf [101]. In an April 2001 meeting with the ICC Secretary General, Maria Cattaui, Broughton tried to raise “the ICC's awareness of the increasingly influential reach of the WHO” [101] and persuade the ICC to seek consultative status from the UN so as to represent corporate interests at the negotiations. A follow-up letter to this meeting suggested that BAT hoped that the ICC could facilitate its engagement with other organisations:

“One of the difficulties we face is a lack of awareness of the responsible face of the tobacco industry. We are working hard to address this situation and I would be interested to know if there are any opportunities for me, or other company members, to represent the ICC in dialogue with multilateral agencies.” [12],[100]

BAT reports show that the company enjoyed high-level access to ICC and illustrate BAT's hopes to impact the ICC's agenda in the context of the FCTC [100],[228].

*Trade unions and other political actors*: TTCs also sought to mobilise organisations that would appear more independent of the corporate sector and the tobacco industry, including consumer groups [120], scientific think-tanks [111], and trade unions [111],[113],[120],[145]. Trade unions were seen as particularly useful in endorsing economic arguments regarding employment (*economic impacts* frame) and in lobbying ministers, so BAT planned to approach international umbrella unions, including the ITGA, IUF [19], and UNITAB [120] to lobby on their behalf. While available documents do not provide evidence about the specific content of BAT's communication with IUF and UNITAB, all three organisations and the German Union of Food and Allied Workers (Gewerkschaft Nahrung-Genuss-Gaststätten) subsequently voiced concerns about the FCTC either at the international level [155] or domestically [113],[155],[156].

BAT used the ITGA particularly intensively in its lobbying efforts to undermine the FCTC. In a 1999 document, Shabanji Opukah (then BAT Head of International Development Issues) explained how he envisaged the relationship between BAT and the ITGA functioning:

“[T]hey are supposed to be working for us at extreme arms length […] It is in cases like this that this whole ITGA relationship should be leveraged for our business advantage and I always aim at doing that and also ensuring that we are in the ITGA's driver's seat” [157].

It was hoped that the ITGA would use discussions and publications about the economic impact of the FCTC for “lobbying goverments [sic] and allies and briefing media on the role of tobacco in the economy” [158] and that it would persuade member states to request an economic impact assessment of the proposed FCTC, thereby delaying the treaty [159] (for a detailed assessment of how and why TTCs believed impact assessments would benefit their interests, see Smith et al. [80],[81]). The ITGA subsequently recruited scientists, think-tanks [158], and UNITAB [160] in support of such efforts and targeted the UN Economic and Social Council [37] and national government and UN representatives in Geneva [39],[114]. The ITGA also commissioned a hostile review of the World Bank's work on tobacco control [17],[161].

*International agencies*: Several international organisations and UN agencies were identified as potential allies, including the UN Economic and Social Council [120],[162], the United Nations Conference on Trade and Development (UNCTAD) [120],[163], the World Customs Organisation [163], the International Labour Organisation [120], the Intellectual Property Organisation [120], the UN Food and Agriculture Organization (FAO) [107],[120], and the WTO [120]. UNCTAD was perceived to be potentially “influential in delivering arguments and messages […] on trade matters” to ministers of employment, agriculture, and trade and to tobacco workers (i.e., in advancing *economic impacts *and *trade* frames) [120]. The ITGA also explored the potential scope for cooperating with the FAO on “academic studies” [139]. Although available documents do not reveal whether such collaboration occurred, both the ITGA [162] and BAT [164] made reference to a subsequent FAO economic impact study which helped BAT draw attention to the economic importance of tobacco production and consumption [164].

Another ally was the International Organization for Standardization (ISO) [165],[166], a worldwide umbrella organisation for national bodies promoting standardisation to facilitate trade [167]. The ISO, represented by a former employee of Imperial Tobacco, attended INB negotiations and was perceived as a valuable source of information and access for tobacco companies [12].

Available evidence suggests that TTCs' efforts to generate concerns among these agencies may have met with some success. For example, documents claim that WHO “met with major resistance, particularly by the tobacco growing countries” when presenting the framework convention proposal to the UN Economic and Social Council in July 1999 [168]. A meeting was subsequently arranged between WHO, WTO, FAO, and UNCTAD to discuss these matters and WHO was asked to consider the economic consequences of a convention [168]. A BAT account of the first session of the INB reported that several UN agencies had voiced concerns about the WHO convention, with the FAO appearing “particularly annoyed” [169].

(4) *Using stakeholder consultation to secure industry participation and delay decisions.*

Ensuring that their concerns were voiced in FCTC discussions and negotiations both enabled the TTCs to address stakeholders and decision-makers and was perceived as a tactic that could delay the FCTC [170]. From the outset, BAT was concerned about the “pace with which the WHO process is moving” [110] and engaged PM, RJ Reynolds, Japan Tobacco, and the VdC in discussions about how to slow it down [110],[171]. Delaying the process was seen as beneficial because it would provide time to promote arguments against the FCTC and allow “governments […] to consider its implications on jobs and money at a time when both are under pressure” [159]. Requesting an impact assessment of the proposals [159], further consultation [161],[172] and additional evidence [162],[173],[174],[175] were all means of delaying the FCTC process [26],[102]. In addition, specific FCTC delegations were encouraged to call for more time [170]. While documents provide no evidence that these tactics were particularly successful, prior to the second meeting of the FCTC working group in March 2000, BAT reported that some governments believed the FCTC process was moving too fast (e.g., Greece and Turkey), some felt insufficient time had been allowed for consultation (e.g., the US), and some merely favoured more time to consider the WHO proposals (e.g., Japan and Russia) [176].

(5) *Using the media.*

Previous research has highlighted how TTCs have successfully used journalists and media outlets to advance their political interests at a national level [177],[178],[179],[180]. Early on in the FCTC process PM noted that “[c]ultivating allies […] in the media is a crucial part of protecting our business” [181], and media channels were subsequently exploited in disseminating several key arguments [49],[138],[158],[182]. BAT targeted media outlets perceived to be supportive of free trade [70], including the Wall Street Journal Europe, where editors gave Broughton the opportunity to write an op-ed during the FCTC public hearings [183]. The Wall Street Journal also published an article by ESEF's Lorraine Mooney [184],[185], focusing on “WHO's misplaced priorities” [186], which drew heavily on the *developing countries* frame. Mooney [186] lambasted WHO for extending its remit to “lifestyle” rather than concentrating on “real” health issues. A 2004 Daily Telegraph article by Bate similarly claimed that WHO's focus on tobacco and obesity signalled that the organisation was “pandering to the desires of its western (especially European) donors, rather than attending to the malnourished millions of Africa and Asia” and suggested that WHO “had lost sight of its mission to save the poorest from easily preventable and cheaply curable diseases” [187].

## Discussion

This paper provides the first comprehensive analysis of TTC tactics to undermine the development of the FCTC. The findings illustrate the variety and complexity of tobacco industry efforts to undermine the FCTC and demonstrate the extent to which TTCs are able to combine and coordinate these approaches on an international stage. In total, our documentary data enabled us to identify eight frames developed by TTCs to advance arguments against the treaty (section A, Results) and five tactics to counter it (section B, Results), which included diverse efforts to effectively disseminate the frames. While all but one frame and one tactic identified in our documentary analysis has been identified in previously published literature concerning the FCTC, our paper provides additional evidence of the extent to which these frames were employed in and adapted to the specific context of the FCTC negotiations and formed part of a collaborative industry strategy to undermine global health governance.

Our comprehensive literature review identified a further six frames and five tactics that were employed by the tobacco industry in their efforts to influence the FCTC. The 14 frames and ten tactics identified in the documents do not consistently match the frames and tactics identified in the literature review which might reflect the primarily national focus of previous analyses of the negotiations. National case studies may be more likely to identify frames which were geared to circumvent the consequences of the FCTC at country level, like the *flexibility* or *education* frame, or which had proven to be particularly successful in the respective national context, like the *personal freedoms* frame. By contrast, the frames identified through our documentary analysis focused more on the global context of the FCTC negotiations, and hence on industry concerns regarding the distinctive scope of this initiative. Such concerns are reflected in attacks on the FCTC's implications for global health and development (as in highlighting claimed threats to producer countries and in describing tobacco control as an issue primarily concerning developed countries) and in depicting the FCTC as an illegitimate expansion of regulatory scope (by allegedly compromising trade agreements, impinging on “good governance” and national sovereignty, exceeding WHO's mandate, and setting an unwelcome precedent). One tactic identified in our literature review for which it is surprising that we did not find further evidence in our documentary analysis is that of TTCs' efforts to counter nongovernmental organisations supporting the development of the FCTC. This may reflect specific ways in which frames were defined during the documentary analysis, but also suggests the difficulties of conducting comprehensive searches in the tobacco industry archives and points to the limitations inherent in tobacco document analysis, particularly given the comparatively restricted availability of documents for this period [188].

All of the frames and tactics we identify as having been used in an FCTC context have roots in strategies previously deployed by TTCs. A review of tobacco industry interference with tobacco control published by WHO in 2008 [43] outlines TTCs' strategies to try to prevent, weaken, and otherwise undermine tobacco control policy. Our findings suggest that in the FCTC context, TTCs focused particularly on trying to prevent what they perceived to be the globalisation of tobacco control. Frames depicting international tobacco control policy as infringing on sovereignty, breaching principles of good governance, a high-income country concern, and exceeding WHO's mandate were identified as particularly suitable for attacking the emerging FCTC. These frames appear as adaptations of those that TTCs had previously used in national and regional contexts (e.g., in debates about EU governance [81] and about the European Commission's competence in tobacco control [12],[189],[190]) and that were now applied to this distinctive emerging global challenge. In contrast, a number of tactics which TTCs employed in other tobacco control contexts (e.g., litigation [43],[178],[189],[190],[191],[192],[193] and political funding [43],[131],[191],[192],[194],[195],[196],[197]), could not be identified as having been used against the FCTC. This might reflect a perception within the industry that some strategies are less suited to a global context. The context-specific adaptation and application of frames reflect the particular opportunities which arise for (re-)framing an issue when policymaking shifts to another venue, i.e., to another institution or level of decision-making [198] and suggests that TTCs can be expected to continue to employ and finesse the same strategies to influence policy across national and international levels.

Considering the success which TTCs enjoyed in framing political and public debates [9] and employing such tactics to counter tobacco control policy at a national level [43], it is perhaps unsurprising that previously used frames and tactics informed TTCs' argumentation in the global governance context of the FCTC. The principal contribution of this paper to the broader literature on tobacco industry efforts to undermine policy lies in its demonstration of how the industry was able to draw on experiences, contacts, frames, and strategies across multiple jurisdictions to develop and deploy a multi-pronged strategy at a global level. The comprehensiveness and scale of the tobacco industry's response to the FCTC suggests that it is reasonable to speak of a “globalisation of tobacco industry strategy” in combating the development of effective tobacco control policies. This highlights the importance of moving beyond national and local case studies of tobacco industry influence to develop a greater understanding of the regional and global dynamics of TTC operations.

The analysis of the TTCs' fight to prevent, then undermine and weaken the FCTC can further serve as a case study for research into how corporate tactics can be employed on a global scale to undermine the development of international initiatives. These findings can inform subsequent efforts to develop tobacco control strategies (including via the implementation of the FCTC). Arguably, however, their greater value lies in their broader relevance to the challenges of developing innovative, international approaches to combat the global burden of NCDs, including via proposals to extend the FCTC governance model to other NCDs. The four leading NCDs (cardiovascular diseases, cancers, chronic respiratory diseases, and diabetes) account for an estimated 60% of all deaths globally and, in contrast to the picture painted by TTCs, 80% of this burden occurs in low- and middle-income countries [199]. These burdens can be viewed as “industrial epidemics” [200], driven by commercial interests and activities (e.g., of food, alcohol, and tobacco corporations), which require new policy approaches to regulating health impacts of the commercial sector [201],[202].

The broader difficulties confronting WHO and other agencies in responding to the escalating global burden of NCDs and the global expansion of the related commercial interests have long been evident; epitomised, for example, by the efforts of the US sugar industry to undermine the Global Strategy on Diet, Physical Activity and Health [203],[204], a comparatively modest initiative [205]. Such difficulties were reiterated in September 2011 at the UN High Level Meeting on NCDs, which stimulated expressions of widespread concern about the influence and impacts of the food and alcohol industries [206],[207]. These concerns underline the importance of finding appropriate approaches for governing interactions between policymakers and the commercial sector, and overcoming any political obstacles to achieving policy coherence between trade and health objectives [208].

Although the arguments and frames analysed above may often be dismissed as spurious or cynical when known to originate with the tobacco industry and considered in the context of public health evidence, they may nevertheless resonate with, and be advanced by, actors with greater credibility, legitimacy, and influence within policy debates. TTCs' assertions that WHO should focus on infectious diseases in developing countries, for example, have much in common with pressures periodically placed on WHO by leading states including the US and UK [209],[210]. The success of the FCTC initiative [211] strongly attests to the importance of protecting (and enhancing) WHO's scope to address NCDs and to develop binding legal instruments [212]. While TTCs' arguments may not have substantially undermined the FCTC, similar arguments may have greater leverage in future debates around strategies to combat NCDs, particularly when advanced by and on behalf of more credible actors.

Research already documents significant similarities between the tobacco industry and a variety of other industries, including alcohol [213],[214], food [214],[215], oil [216],[217], chemical [216],[217],[218], and pharmaceuticals [216],[218], which further underlines the potential relevance of the findings in this paper for issues beyond tobacco control. This is particularly true of alcohol, where the prospect of a Framework Convention on Alcohol Control is increasingly being discussed by public health academics [5],[219]. The findings could be used to help identify cross-industry counter arguments and dissemination techniques, including any instances of the same third parties, scientists, and media outlets being employed. Indeed, although TTCs do not seem to have been particularly successful in persuading other sectors that the FCTC was likely to set a precedent for other areas (i.e. in advancing the *precedent* frame), this could change as interest develops in the applicability of the FCTC model for other areas. Given that transnational corporations appear to be well placed to jointly develop counterattacks on proposals relating to global health governance, those who aim to develop comprehensive, international solutions to these health problems need to be aware of knowledge transfer and cross-sectoral collaboration. The lukewarm response to the recent declaration of the UN High Level Meeting [220],[221] attests to the importance of such approaches, illustrating the scale of political obstacles involved in combating commercial interests beyond the tobacco industry. This is starkly evident in the disparity between the declaration's strong focus on supporting FCTC implementation and the modest voluntary strategies envisaged for NCD regulation more broadly [222].

## Supporting Information

Alternative Language Abstract S1 German translation of the abstract.
(DOC)

## Abbreviations

BAT: British American Tobacco
CSR: corporate social responsibility
ESEF: European Science and Environmental Forum
FAO: United Nations Food and Agriculture Organization
FCA: Framework Convention Alliance
FCTC: WHO Framework Convention on Tobacco Control
IAA: International Advertising Association
ICC: International Chambers of Commerce
INB: International Negotiating Body
ISO: International Organization for Standardization
ITGA: International Tobacco Growers' Association
IUF: International Union of Food, Agricultural, Hotel, Restaurant, Catering, Tobacco and Allied Workers' Associations
MBD: Mongoven, Biscoe & Duchin
NCD: non-communicable disease
PM: Philip Morris
TTC: transnational tobacco corporation
UNCTAD: United Nations Conference on Trade and Development
UNICE: Union of Industrial and Employers' Confederations of Europe (now BusinessEurope)
UNITAB: International Union of Tobacco Growers
VdC: Verband der Cigarettenindustrie
WHO: World Health Organization
WTO: World Trade Organization
