# Supplementary material for: Global Health Governance and the Commercial Sector: A Documentary Analysis of Tobacco Company Strategies to Influence the WHO Framework Convention on Tobacco Control
Source: PLoS Med. 2012 Jun 26;9(6):e1001249. doi: 10.1371/journal.pmed.1001249 (PMC3383743; doi:10.1371/journal.pmed.1001249)
Supplement: Alternative Language Abstract S1 — German translation of the abstract. (DOC) [file pmed.1001249.s001.doc]

Globale Gesundheitspolitik und kommerzieller Sektor: Strategien der Tabakindustrie zur Einflussnahme auf die WHO Rahmenkonvention zur Tabakkontrolle

Zusammenfassung

Hintergrund: Durch die erfolgreichen Verhandlungen der WHO Rahmenkonvention zur Tabakkontrolle (FCTC) hat die Weltgesundheitsorganisation eine bedeutsame Innovation in der globalen Gesundheitspolitik eingeleitet und geholfen, die internationale Tabakkontrolle zu transformieren. Dieser Artikel bietet den ersten umfassenden Überblick über die facettenreiche Tabakindustrie-Kampagne, welche zum Ziel hatte, das Rahmenabkommen zu unterwandern.

Methodik und Ergebnisse: Der Artikel gründet sich primär auf die Analyse von internen Tabakindustriedokumenten, welche aufgrund von Gerichtsverfahren für die Öffentlichkeit zugänglich sind. Diese Dokumente wurden mit öffentlichem Datenmaterial über den FCTC Prozess und von Internetseiten massgeblich beteiligter Organisationen trianguliert. Der Artikel beinhaltet andererseits einen umfassenden Review der wissenschaftlichen Literatur über den Tabakindustrie-Einfluss auf die WHO Rahmenkonvention. Die Ergebnisse zeigen, dass die strategische Antwort der Industrie auf den Vorschlag des Rahmenabkommens zweigeteilt war. Einerseits wurden Argumente und Bezugsrahmen entwickelt, mit dem Ziel, das Rahmenabkommen in Frage zu stellen. Diese beinhalteten Argumente, dass das Abkommen negative Konsequenzen für die Wirtschaft habe, die Tabakkontrolle ein von entwickelten Ländern vorangetriebes Gesundheitsthema sei, mit Handelsabkommen, verantwortungsbewusster Regierungsführung und nationaler Staatshoheit in Konflikt stehe, nicht dem WHO Mandat entspreche, einen Präzedenzfall für die Regulierung anderer Politikbereiche setze, und durch Massnahmen der sozialen Verantwortung von Unternehmen ersetzt werden könne. Andererseits setzte die Industrie verschiedene Taktiken ein, um diese Argumente zu verbreiten und ihre Wirkung zu erhöhen. FCTC Delegationen und relevante politische Akteure wurden als Zielgruppe ins Auge gefasst; verschiedene Bündnispartner (z.B. Vertreter der Massenmedien und Wissenschaftler) wurden zur Mitarbeit an der Kampagne gewonnen und Anhörungen von Interessensgruppen wurden genutzt, um Entscheidungen zu verzögern und die Beteiligung der Industrie am Prozess sicher zu stellen.

Schlussfolgerungen: Die Bemühungen der Tabakindustrie, die Rahmenkonvention zu unterwandern, waren umfassend und demonstrieren die globale Anwendung von Taktiken, welche die Tabakindustrie in der Vergangenheit in der Vergangenheit im nationalen Kontext einsetzte. Die Tabakindustrie argumentierte darüber hinaus spezifisch gegen die Rahmenkonvention als eine Schlüsselinitiative der globalen Gesundheitspolitik. Ein Bewusstsein über solche Strategien hat das Potential, den Erfolg weiterer Tabakindustrie-Bemühungen bezüglich der Implementation der Rahmenkonvention zu verringern und die Entwicklung von zukünftigen, vergleichbaren Initiativen der globalen Gesundheitspolitik zu unterstützen.

Schlüsselwörter: Tabakindustrie, politische Entscheidungsfindung, unternehmerischer Einfluss, global
